# Some of the Less Frequently Recognised Affections of Joints

**Published:** 1892-06-25

**Authors:** 


### SURGERY.

### SOME OF THE LESS FREQUENTLY RECOGNISED AFFECTIONS OF JOINTS.

There are some joint affections which have only been recognised and thoroughly described of late years—at any rate, in English works on surgery; and we propose in this paper briefly to refer to some of them.

That the joints are affected in locomotor ataxia is of course well known, but the characters of this joint lesion are not perhaps so well recognised, especially as it often occurs before any inco-ordination of movement is present in the limbs. It usually begins with great swelling, not only of the joint, but of the whole limb; and then as the swelling around the joint subsides, we find the joint distended with synovial effusion. But the great point of importance in the diagnosis of this affection in the early stage is the painless character of the effusion, and of the general swelling in the limb. After the onset of the effusion, which may to a large extent be absorbed, the ligaments become relaxed, allowing of undue movement in the joint, and the articular surfaces wear away. This wearing away of the articular surfaces is, perhaps, one of the most characteristic features of the disease, and dislocation of the joint may occur either in the earliest stage, during the great effusion from relaxation of the ligaments, or during a later stage from wearing away of the ends of the bones. Sometimes the disease is symmetrical.

But all the cases do not begin with this extreme swelling of the joint. Some begin more like cases of osteo-arthritis, with gradual enlargement and osteophytic growth at the articular ends of the bones, and with grating in the joint from the first. And these osteophytic growths may also be present in cases where there is effusion. We have lately seen a Charcot's joint in which there was a large detached osteophyte in the joint with others still attached, and considerable effusion into the synovial cavity. The patient had undoubted locomotor ataxia with ocular paralysis. In another case we have seen the articular ends of the bones so covered with large osteophytic growths, that *per se* the joint could not be distinguished from osteo-arthritis. In this case there was also ataxia and perforating ulcers of both feet. However, these osteophytes are the exception, not the rule, in Charcot's disease, and the usual absence is a point of distinction often urged as separating this joint lesion from osteo-arthritis. Osteo-arthritis is not usually an acutely painful affection, whereas Charcot's joints are painful from first to last. They are apparently due to defective trophic innervation. In osteo-arthritis again we do not get such great effusion into the joint at the beginning, or such lateral mobility at a later stage.

The next joint affection to which we wish to direct attention is tubercular dropsy of joints, as it has been called, or hydrops tuberculosis. This form has been described by König. Sometimes we see an ordinary "white swelling"—pulpy tubercular joint lesion, begin with moderate synovial effusion. This joint affection is to pulpy tubercular disease of the joint what tubercular pleural effusion is to phthisis, except that in the knee joint the ordinary tubercular disease generally begins in the synovial membrane, and why in the one case it should cause effusion only, and in the other infiltration of the joint structures, is not clear. That König's hydrops tuberculosis is tubercular has been proved by microscopic examination of the synovial membrane. That sometimes, however, obstinate effusion has gone on to ordinary white swelling suggests that perhaps in this hydrops tuberculosis it is merely that the tubercular affection in its early stage fails to infiltrate the other structures of the joint, *i.e.*, that it is a difference in degree rather than in character.

Clinically, we find movement in the joint painless, and König says he has sometimes felt a fibroid mass resembling a tumour at the lateral reflexion of the synovial membrane. This fibroid mass sounds more like a syphilitic joint than a tubercular one, and we propose now to consider such lesions.

English text-books on surgery say very little about syphilitic joint diseases, and it is impossible to get from them sufficient information on the subject. Mr. J. Hutchinson, jun., has, however, recently brought present knowledge on the subject to a focus in an admirable lecture at the College of Surgeons.*

Of the synovitis with effusion occurring in the secondary stage we need not write much, as that is the most commonly recognised and described affection. Of the later manifestation the "syphilitic pseudo white swelling," described by Richet, is the commonest form. As the name implies, it clinically resembles tubercular pulpy disease of joints, as there is a uniform thickening of the synovial tissues, but unlike the even doughy swelling of tubercular disease, we find generally firm nodules in the synovial membrane or capsule. It is very painless, and there is very slight interference with mobility, and often several joints are affected, sometimes symmetrically. The affection begins insidiously, and the cases improve readily with antisyphitic treatment, but if neglected, may lead to fibrous anchylosis and contraction.

Another form of the disease consists in gummatous infiltration around the joint, with effusion into its cavity. Usually the gummata do not bear down into the joint, but through the skin. This form is one, therefore, easily recognised. The most difficult form to diagnose is that in which a gumma forms under the periosteum at the articular end. Such cases closely resemble sarcoma, and the resemblance is further increased by the presence of effusion into the joint, and they do not always clear up with iodide and

* *Brit. Med. Journal*, April 16th, 1892.

mercury even when given in large doses. The severe aching pain at night present in these cases is, however, unlike sarcoma. Hutchinson relates the case of a man who had such a growth in the lower end of the femur, which even on preliminary exploration so resembled a sarcoma, that the limb was amputated. Hutchinson says: "It was only after careful microscopic examination had been made that the nature of the disease was fully established."*

* "Trans. Patholog. Soc.," 1887, p. 288; and see also Howard Marsh's "Book on Diseases of Joints."

